# Pre-Computing Batch Normalisation Parameters for Edge Devices on a Binarized Neural Network

**DOI:** 10.3390/s23125556

**Published:** 2023-06-14

**Authors:** Nicholas Phipps, Jin-Jia Shang, Tee Hui Teo, I-Chyn Wey

**Affiliations:** 1 Engineering Product Development, Singapore University of Technology and Design, Singapore 487372, Singapore; 2 Department of Electrical Engineering, Chang Gung University, Taoyuan City 333, Taiwan

**Keywords:** batch normalisation, binarized neural networks, convolutional neural networks, inference, edge devices

## Abstract

Binarized Neural Network (BNN) is a quantized Convolutional Neural Network (CNN), reducing the precision of network parameters for a much smaller model size. In BNNs, the Batch Normalisation (BN) layer is essential. When running BN on edge devices, floating point instructions take up a significant number of cycles to perform. This work leverages the fixed nature of a model during inference, to reduce the full-precision memory footprint by half. This was achieved by pre-computing the BN parameters prior to quantization. The proposed BNN was validated through modeling the network on the MNIST dataset. Compared to the traditional method of computation, the proposed BNN reduced the memory utilization by 63% at 860-bytes without any significant impact on accuracy. By pre-computing portions of the BN layer, the number of cycles required to compute is reduced to two cycles on an edge device.

## 1. Introduction

It is becoming increasingly common to find Neural Networks on edge or mobile devices. Two examples of these are objection detection and natural language processing. Quantized Neural Networks are increasingly used as a solution to this problem, and BNNs are one of these solutions [1].

While BNNs have a reduced memory footprint compared to an equivalent CNN, full-precision values are still necessary during other operations within the network. This presents an opportunity to reduce memory requirements further. It is especially important since Static Random-Access Memory (SRAM) scaling on newer process nodes has been almost negligible at 5.5% [2,3] compared to logic transistors at 67%. There are a few methods to accomplish this, the simplest being a simple reduction in the precision of the number representation (i.e., 32-bit to 16-bit) post-training, incurring a minor penalty in accuracy.

In this paper, the solution presented reduces memory utilisation and computational complexity on edge devices. A linear transformation is applied to a set of layers, which allows for the aspects of a BNN to be pre-computed on a high-performance device prior to quantization and export. This reduces the loss in accuracy from quantization and simplifies the instructions necessary, reducing the computational load.

### 1.1. Binarized Neural Networks

A BNN is a reduced precision CNN, boasting a smaller overall model size and a faster classification at a minimal cost to accuracy. Its weights have been reduced to two differential states, +1 and −1 in real terms, transformed to 1 and 0 once encoded. However, not all layers must be fully binarized to qualify as a BNN. For example, binarizing a network’s input would approximate away the granular information—hampering the ability of the network to train on and discern finer features. In addition, Larq [4] found that accuracy can be improved by not binarizing the final layer.

**Figure 1:**
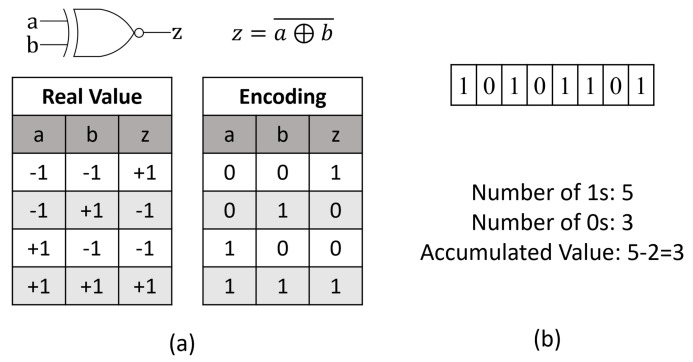
Binarized MAC Operation. (**a**) Use of an XNOR to replace a full-precision multiplication unit (**b**) Use of Popcount in Binarized Accumulation.

A BNN consists of Binarized Convolution, Fully-Connected (FC), Pooling, and BN layers [5]. It uses the Sign Function to binarize output feature maps for the next Convolution/FC layer, represented in Equation (2) [6]. In a traditional CNN, a Multiply-Accumulate (MAC) operation can be represented as Equation (1). In a BNN, it is replaced with an Exclusive NOR Gate (XNOR), and Popcount, is an instruction that returns the number of 1s in a group of bits. With the total number of bits and 1s, an accumulated value can be found in Figure 1. It is important to note that the input layer does not binarize the inputs and only the weights, preserving the features of the input. However, as [7] notes, the input layer is generally smaller than the subsequent layers.

(1) $$c_{m}=\sum_{n=1}^{N}x_{n}\times w_{m},n+b_{m}$$

where *n* represents the number of input channels, *m* represents the number of output channels, *c* is the output of the Convolution layer, *x* is the input, *w* is the weight, *b* is the bias of the channel.

(2) $$x^{b}=Sign\left(x\right)=\left\{\begin{array}{ll} +1 & \mathrm{if}\;x\geq 0,\\ -1 & \mathrm{if}\;\mathrm{otherwise}, \end{array}\right.$$

where $x^{b}$ is the binarized weight/activation and *x* is the real-value weight/activation.

### 1.2. Batch Normalisation

BN [8] is necessary for a BNN, providing stability to the network from its extreme quantization. However, it requires full-precision values and different mathematical operations to compute even during inference. Requiring multiple full-precision hardware logic units to be implemented, which take up a larger area. This makes BN a prime target to reduce hardware complexity and speed up overall inference.

During inference, BN can be split into two constituent parts; Normalise, and another operation known as ‘Scale and Shift’. Normalise, represented in Equation (3), inherits its statistical parameters from the training set. These are the training set’s mean (μ) and variance ($\gamma^{2}$). During training, these values are updated to maintain an output feature map with a μ close to 1 and a $\sigma^{2}$ close to 0.

Scale and Shift, represented in Equation (4), learn its parameters through Backpropagation. These are the scaling factor (γ) and the offset factor (β). Both Equations (3) and (4) combine to form the BN inference Equation (5) [8]. Equation (6) is a recent update from TensorFlow [9] which changed moving variance ($\sigma^{2}$) to moving standard deviation (σ) giving the learnt parameters greater weight over the inherited parameters.

(3) $$V_{i}^{\prime }=\frac{V_{i}-\mu }{\sqrt{\sigma^{2}+\epsilon }}$$

(4) $$V_{o}=\gamma \times V_{i}^{\prime }+\beta$$

(5) $$V_{o}=\gamma \times \frac{V_{i}^{\prime }-\mu }{\sqrt{\sigma^{2}+\epsilon }}+\beta$$

(6) $$V_{o}=\gamma \times \frac{V_{i}^{\prime }-\mu }{\sqrt{\sigma +\epsilon }}+\beta$$

where μ is the moving average, $\sigma^{2}$ is the moving variance, σ is the moving standard deviation, γ and β are the learned scale and shift parameters, $V_{i}$ is the input to the BN layer, $V_{i}^{\prime }$ is the intermediate output from the normalise operation, $V_{o}$ is the output of the BN layer.

While CNNs are computationally demanding, BN is essential to a BNN [10]. Different mathematical operators are required to compute BN during inference, and implementing additional logic units affects chip area utilisation and has additional static power dissipation. These may become relatively expensive as the additional full-precision logic units cannot be used by the binarized layers, unlike a CNN.

### 1.3. Implementing Batch Normalisation on Hardware

Beyond quantizing Full-Precision parameters, previously adopted measures to reduce hardware complexity and memory consumption were as follows; on Intel’s [11] accelerator, merged parameters across layers to pre-compute new parameters reducing memory requirements and compute complexity. In this work, the same MAC Processing Element (PE) is reused and can be seen in Equations (7) and (8). However, this only accounts for the Scale and Shift portion (Equation (4)) of the BN process and not the more computationally complex normalise portion (Equation (3)).

(7) $$V_{o}^{\prime }=V_{i}\times w+\left(b-\mu \right)$$

(8) $$f\left(V_{o}\right)=f\left(V_{o}^{\prime }\times \gamma +\beta \right)$$

In Cornell’s [12] accelerator, a linear transformation is used to reduce pre-compute certain elements of the BN process. A linear transformation is applied to the inference BN equation to reduce the computational burden on the hardware accelerator. Two constants *k* (Equation (10)) and *h* (Equation (11)) are used to simplify the computation process. This results in the Linear Equation (9). Unlike [11], the simplification adopted exists solely within the BN layer. This means that the bias component is dropped, causing a 1% increase in the test error rate. While seemingly insignificant, this is important since BNNs generally perform slightly worse when compared to similar CNNs [6].

(9) y=kx+h

(10) $$k=\frac{k}{\sqrt{\sigma^{2}+\epsilon }}$$

(11) $$h=\beta -\frac{\mu \gamma }{\sqrt{\sigma^{2}+\epsilon }}$$

where *k* and *h* are the arbitrary constants defined in Equations (10) and (11).

Unlike the previous two works discussed, ref. [13] proposes an accelerator that focuses on BN. This work has two contributions; the first is a reduced complexity BN layer named LightNorm. Furthermore, the second is a hardware accelerator that implements the LightNorm layer. The accelerator consists of hardware for both Forward Propagation and Backward Propagation, accelerating both Training and Inference. This work uses approximated arithmetic and tensor grouping with the same exponents to reduce the number of computations required. While the contributions from this work are significant, the use of Floating Point (FLP) representation limits adoption, especially on low-complexity edge devices.

To evaluate these, a reference point is necessary. In addition to pre-existing work from [6,11,12,13,14,15] used as a basis, training is performed with the aid of TensorFlow [16] and a BNN extension, Larq [4]. Network weights and parameters are then exported for further processing using the methodology elaborated in the subsequent section.

### 1.4. Resource-Constrained Edge Devices

Improving performance on edge devices requires tailoring implementations around constrained resources. With the expected SRAM scaling issues going forward, memory operations are a key area to tackle. As training is performed on more capable general-purpose hardware, optimising for edge devices focuses primarily on inference.

#### 1.4.1. TensorFlow Lite

TensorFlow Lite [17] is a solution that uses FlatBuffers [18] and custom operators to reduce latency, memory, and power usage. It has a derivative focused primarily on Microcontrollers, TensorFlow Lite Micro [19], with support for Cortex Microcontrollers with CMSIS-NN [20]. It allows model conversion through the TensorFlow Lite Converter.

#### 1.4.2. Larq Compute Engine

Larq Compute Engine [21] is a similar solution to TensorFlow Lite that focuses primarily on BNNs. It supports conversion from Larq-based BNN models to a .tflite-based output.

Amongst the various strategies employed, operation fusing is very useful. In TensorFlow Lite, layers are fused, in the order Conv2D, BatchNorm, and ReLU. By fusing multiple layers, the singular resultant operation can be computed faster with fewer parameters. Furthermore, in Larq Compute Engine, layer fusing is also supported. However, due to the nature of a BNN, these are in a different order; namely, QuantConv2D, ReLU, and BatchNorm.

There are other strategies involved such as Integer-Arithmetic-Only Inference [22], Quantization, Pruning, and Clustering. When combined together, these are an essential package for running inference on edge devices.

### 1.5. Further Discussion

The next section will discuss a proposal to reduce hardware complexity requirements. These can be split into two general methods:

1. Parameter Quantisation: Reducing the precision of real-valued parameters comes at a minor cost to accuracy but can significantly reduce memory usage.
2. Layer Grouping and Computation Re-ordering: Equations can be simplified by grouping layers and re-ordering computations to use fewer logic units. This can be performed post-training and prior to loading the parameters on an edge device. Since equations are simplified, the total number of parameters can be reduced, reducing memory usage.

The final section discusses the performance impact of these methods. Both will be evaluated in terms of accuracy loss, memory, and logical operation reduction.

## 2. Specification and Proposal

This section discusses the Design Methodology and Proposal. In-depth specifications on the bit representation and the reference network are also presented in this section.

### 2.1. Methodology

#### 2.1.1. Processing Parameters

**Figure 2:**
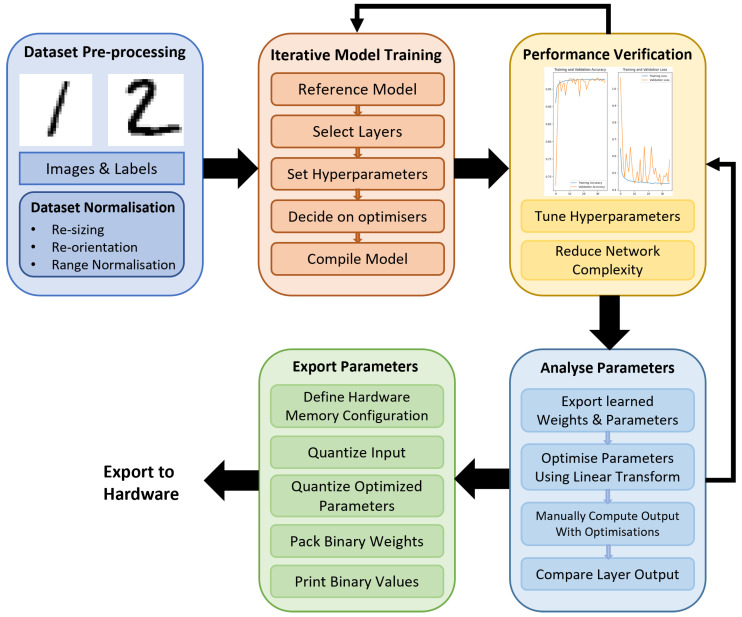
Model processing flow: input dataset from MNIST [23].

Figure 2 details the general process that was taken from the initial stages of training to specific steps taken before exporting the weights and parameters. Dataset pre-processing is a process that normalises the dataset for use within the network. In the case of a BNN, the value range is normalised between −1 and +1 for the best performance.

Model training is an iterative process that consists of the training, verification, and tuning of the network based on the results. Training convergence and the target model specifications will eventually be achieved by tuning the hyperparameters and the learning optimisers.

To implement the proposal, verification of the correctness of the output is necessary. To do this, layer outputs are extracted from the trained model with the same test input. This is then compared against the modified parameters once computed. Essentially, the model will be running on TensorFlow [16] and Larq [4] as a 32-bit Floating-Point (FLP) benchmark.

#### 2.1.2. Grouped Layer Operations

Layers are grouped together and treated as a single entity to allow for the re-arrangement of element-wise mathematical operations. This allows for the simplification of the mathematical operations during full-precision computation. This means that the number of different logical processing units can be reduced on hardware. There is also an opportunity to reduce the number of exported parameters saving on total memory utilisation.

**Figure 3:**
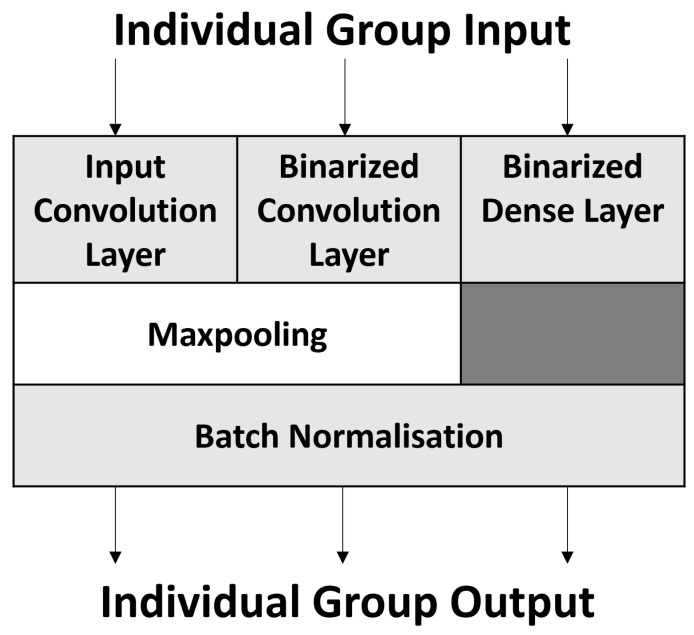
The three different grouped layers. Computations within the same group are re-arranged.

Figure 3 highlights the three different grouped layers. The first and second groups are the Full-Precision and Binarized Convolution groups, consisting of the Convolution layer, Max-Pooling, and BN. Max-pooling is not used in all cases, and removing it would be inconsequential to the output in the proposed changes. The third group consists of a Binarized Dense layer and BN.

The Full-Precision Convolution group demonstrates that the proposed method applies not only to a BNN but also to a CNN. While the binarized groups are specifically for use within a BNN, the proposed method applies for dense layers in a CNN similarly to the relationship between the first and second group.

Grouping these layers together allows operations from other layers within the same group to be moved. Once operations have been shifted between layers, it will become easier to simplify the process. By employing this strategy, parts of a layer can also be computed on a high-performance machine reducing the computational burden on an edge device. Section 2.3.2 will cover this in greater detail.

### 2.2. Reference Network

#### Pico MNIST BinaryNet

This network is a stripped-down model of Larq’s [4] BinaryNet that uses the MNIST [24] dataset. The training was performed over 500 epochs. An accuracy of 96.11% was achieved during post-training inference. The convolution layer window uses a 3×3 window with a stride of 1. Max-pooling is performed with a 2×2 window with a stride of 2×2. Table 1 is a representation of all layers within the network with the number of weights and parameters. BN parameters take up 39.5% of the total memory footprint.

A second copy of the same network is used without bias and the scaling factor (γ). The training was performed over 500 epochs. An accuracy of 94.58% is achieved during inference. It is approximately 25% smaller in size without these two parameters. This would serve as a comparative basis for the same network size in the latter section.

In this subsection, it can be seen that smaller networks have a greater proportion of memory consumed by the BN parameters. Reducing these will allow networks to run on resource-constrained devices. Hardware designs using these networks are also much more flexible in lowering overall power consumption or maintaining a low inference latency.

### 2.3. Proposed Quantization Method

**Figure 4:**
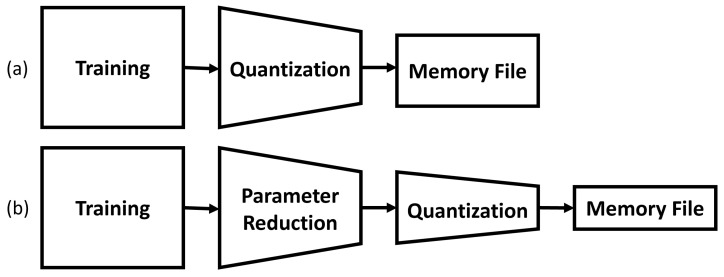
(**a**) Typical Post Training Quantization. (**b**) Proposed Quantization Method.

The methods used for operation simplification are further quantizing parameters or re-arranging operations to reduce the necessary logic units. The former is much simpler to implement, and discussions on the topic will be light. The latter will take up a larger proportion of the discussion. These are completed prior to exporting the parameters to edge devices. Figure 4 demonstrates the proposed process flow of exporting parameters to edge devices.

#### 2.3.1. Parameter Quantization

By default, Keras [25] trains and stores a network’s parameters in 32-Bit FLP. However, since Full-Precision values in a BNN are much fewer, Fixed-Point (FiP) representation is used. Both are represented differently, with the FLP data format using IEEE-754 [26] and FiP format used for the proposed method. (FiP) values can also be computed with only an integer arithmetic compute unit.

The FLP data format is expressed as {S,E,M} where S represents the sign-bit, E represents the exponent bits, and M represents the mantissa bits.

**Figure 5:**
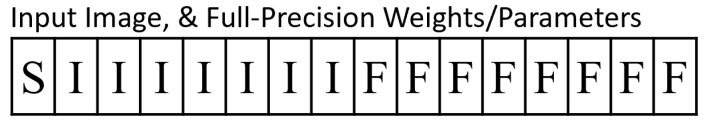
16-Bit FiP representation.

The FiP data format is expressed as {S,I,F} where S represents the sign-bit, I represents the integer bits, and F represents the fractional bits. Figure 5 is an example of a 16-Bit FiP Representation that uses the {1,7,8} configuration. Fixed point values are exported with the aid of [27].

In the latter portion of the work, different bit widths are used to find the relationship between accuracy differences and memory usage. This can be useful when designing custom configuration and hardware for running BNNs. This is performed post-training before quantising parameters to reduce the quantization effect.

#### 2.3.2. Reduction of Arithmetic Operations

The method proposed in this section reduces the five parameters (b,μ,σ,γ,β) in either convolution group to three. This is possible by merging several parameters into two new constants, *j* and *k*.

Equation (12) is comprised of two parameters from two layers, bias from Equation (1) and μ from Equation (3). The resulting constant is computed during convolution and can be represented as Equation (14). The BN layer traditionally requires multiple mathematical operators and would be simplified to a linear equation with fewer parameters. This is performed with constant *k* from Equation (13), resulting in Equation (15).

(12) j=b−μ

(13) $$k=\gamma \times \frac{1}{\sqrt{\sigma +\epsilon }}$$

(14) $$c_{m}=\sum_{n=1}^{N}x_{n}\times w_{n}+j$$

(15) z=c×k+β

where *x* is the input, *w* is the weight, *b* is the bias of the channel, μ is the moving average, σ is the moving standard deviation, γ, and β are the learned BN scale and shift parameters, *j*, and *k* are the arbitrary constants defined in Equations (12) and (13), where *n* represents the number of input channels, *m* represents the number of output channels, *c* is the output of the convolution/max-pooling layer, *z* is the output of the BN layer.

**Figure 6:**
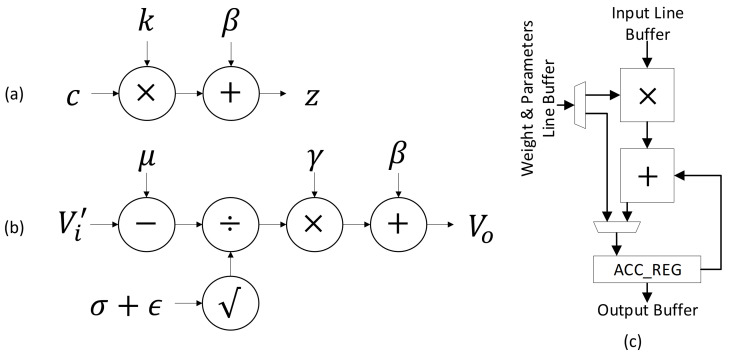
Hardware-Implemented Batch Normalisation. (**a**) Compute Flow for the Proposed Linear Transformation (**b**) Compute Flow on a Traditional Batch Normalisation. σ and ϵ is computed together. (**c**) A generic MAC PE that runs the transformed equation.

Figure 6 demonstrates the proposed compute process compared to the traditional compute flow if implemented on Hardware. There are several advantages to reducing the number of element-wise mathematical operators, which are as follows:

1. The process uses 32-Bit FLP to compute a part of the equation prior to being exported. This reduces the quantization effect from the exported parameters.
2. By going from five parameters to three, the memory requirements are lowered. This reduces the amount of memory needed on-chip.
3. Additional arithmetic units to compute division or square root operators are no longer needed. These take up a greater area and more clock cycles to compute fully.
4. The use of a linear equation means that the logic block can be re-used in a CNN for a MAC operation. Figure 6c demonstrates a generic MAC PE unit.

### 2.4. Proposal Discussion

The proposed quantization method is performed before quantization. This would provide benefits with no real downsides since the model’s weights and parameters are fixed during inference.

In theory, going from five full-precision parameters to three reduces the memory footprint of a BN layer by 40%. This also extends to the hardware; reducing the number of arithmetic operations from five to three reduces the necessary instructions allowing simpler hardware to run BN.

In the case of Application Specific Integrated Circuit (ASIC), a simpler hardware design would require far less area and potential savings in other areas. Since the proposed method is a linear equation, the same MAC PE in Figure 6c can be reused for convolution operations.

## 3. Results

This section examines the relationship between accuracy and memory consumption on the network. The aim is to attain as high an accuracy as possible with the smallest possible memory footprint. There are several limitations in comparing the efficacy of the proposed changes, which arise from the limitations of a BNN.

In this work, a relatively small dataset, MNIST, has been used as a test vehicle to validate the proposed method to accommodate the limited computation resources. Table 2 presents different network configurations with their corresponding parameter precision, data format, and memory utilisation. All configurations are tested with 10,000 test samples. Pico(A) is the network found in Table 1 with five parameters reduced to three. Furthermore, Pico(B) is a derivative trained without bias and γ with the same memory footprint after Pico(A) has gone through a linear transformation.

It is important to examine the impact of precomputing on the BN layer, with and without quantization. In Pico(A), 32-Bit FLP refers to the configuration used in TensorFlow/Larq. Furthermore, 32-Bit FiP refers to the precomputed parameters being used without quantization. Pico(B)’s 32-Bit FLP is provided as an additional point of comparison with the same memory footprint.

To understand the relationship between the selected bit significance and its impact on accuracy, inference was run with the parameters quantized to different configurations. Two main factors impact the Pico Networks, the size of the integer bits representation and the fractional bits precision.

From Table 3, the integer bits representation needs at least 7 bits of precision to fully represent the full number range of the output feature maps and the parameters. In the proposed design, going below 7 bits will cause values to saturate. Fractional bits precision affects the ability of the layer to fine-tune the normalisation process. With the Pico Networks, a precision of 6 bits is an adequate amount with minimal impact.

Looking at the normalised accuracy and memory utilised, there are no observable differences between both Pico 32-Bit FLP and FiP implementations. This would indicate that the proposed quantization method does not incur an accuracy penalty. However, as seen in Pico(B), excluding bias and γ comes at a cost to accuracy.

Taking it further, 16-Bit and 14-Bit FiP demonstrate no significant changes in the ability of the network to infer on the test dataset when quantizing after the pre-compute process. In this particular case, 14-Bit Pico(A) FiP would be the ideal implementation to export to an edge device. At a memory utilisation of 63% compared to the original, with a negligible loss in accuracy, it would be a good choice for a memory-constrained device.

**Figure 7:**
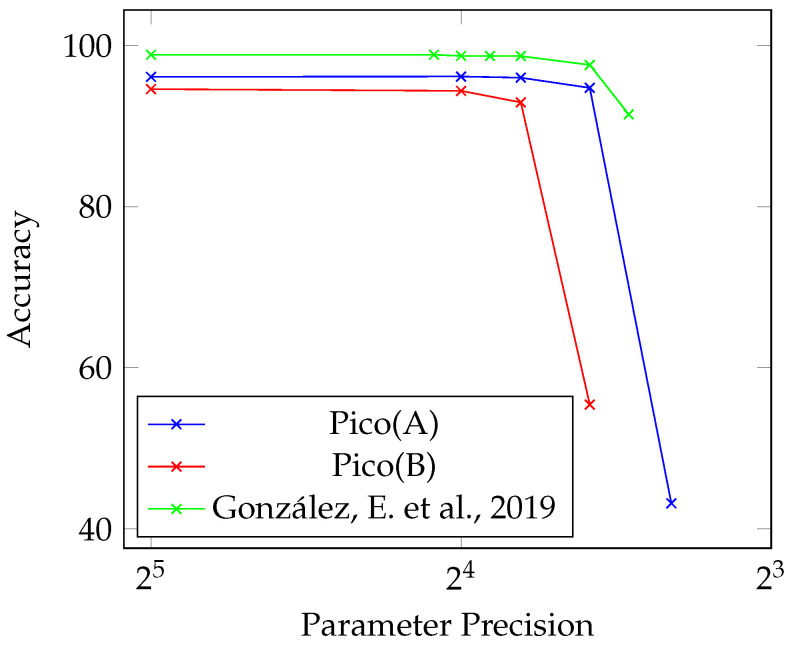
Accuracy drop from parameter quantization, ref. [31].

Figure 7 is a demonstration of the relationship between quantizing parameters and the ability of the network to infer accurately. As it is difficult to assess between the different network configurations, the main takeaway here is that it is important to understand the relationship between the full number range representation and the quantization applied.

When comparing against CNN implementations, the key finding is that huge reductions in the memory footprint can be attained with a minor reduction in accuracy. When comparing against [31], a BNN has the same drop-off in accuracy when quantizing when compared to a typical CNN.

When comparing the drop-off in accuracy between Pico(A) and Pico(B), it is clear that additional learned parameters help to stave off the drop in accuracy from quantization. With the proposed method, there is no additional memory or computations necessary when incorporating these parameters. This happens as a result of pre-computing parts of the process.

With an accuracy drop within 3%, total network memory utilisation is reduced by up to 39%. When implementing these on resource-constrained edge devices, these savings are significant.

To demonstrate a more practical use case, the proposed work was tested on larger production-ready networks. ImageNet [32] was tested with both a CNN and BNN implementations. Table 4 is an extension to the results found earlier in Table 3. The typical method uses 32 FLP and the proposed method adopts 32 FiP.

In [33,35], the four BN parameters are used, and these are simplified to three parameters saving on memory. Similar to Pico(B), in [34], γ is not used reducing the possible savings in memory to zero. However, since portions of the BN layer are pre-computed, the number of cycles needed on an edge device to compute the layer is still reduced.

Bias is the only parameter not found in all three networks. In a Full-Precision Convolution layer, as in Equation (1), it adds an additional parameter per channel and a clock cycle per output feature. While its impact is small, with an additional 1% of accuracy in MNIST and CIFAR-10 [12], it still has an impact. In the proposed work, because parameters are merged across layers, having an additional bias parameter has no additional impact on memory utilised or clock cycles.

**Figure 8:**
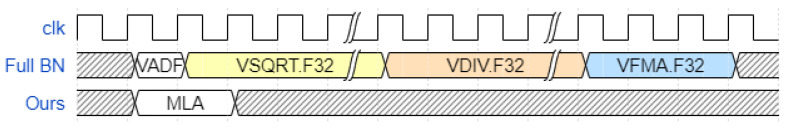
Instruction sequence of the conventional at 32 cycles and proposed method at 2 cycles on a Cortex-M4. Instruction timing from [36]. VADF is a shortened form for the VADD.F32 instruction on a Cortex-M4. Both VSQRT.F32 and VDIV.F32 takes 14 cycles to compute.

The benefits of using the proposed method goes beyond memory utilisation. Since time-consuming instructions are pre-computed, the computational complexity for edge devices is reduced. In Figure 8, a Cortex-M4 running a full 32 FLP BN inference is compared against the proposed method.

A Cortex-M4 was chosen in this example since it has a FLP Unit present which is necessary to compute FLP. Reducing the number of cycles required from 32 to 2 cycles is a significant difference, but it must be noted that this example does not include other instructions (e.g., store/load) of a typical operation.

For practical use, the entire operation would usually have additional instructions, such as fetch and store. Furthermore, it would also be affected by bottlenecks in other areas, such as the memory bus. Whilst this would narrow the difference in the number of cycles required to compute a feature, specialised hardware, such as an ASIC-based accelerator, would be able to fully utilise the benefits of the proposed work.

BN takes up a small portion of the network, and it is often overlooked. In [33], the BN parameters account for roughly 1% of the total model size and it requires a larger variety of instructions to compute. The proposed method has shown that improvements made in this layer are still relevant for edge devices.

## 4. Conclusions

Before exporting the weights and parameters, the proposed improvements to the BNN make it possible to utilise it on much smaller edge devices, such as a microcontroller. A minimal loss in accuracy is possible since computationally complex operations are pre-computed on a host device at full precision prior to quantization.

If precision needs to be preserved, operations on devices without a FLP unit can be made simpler without quantization. Devices without onboard memory can be used with parameter reduction and quantization for minimal cost. To customise for particular applications, these features can also be combined with or added to other solutions.

However, it is also important to verify the peculiarities of the network and the hardware employed. Saturation and overflow are issues that would occur if the integer bits portion is over-quantized. Additionally, the fractional bits allow for fine-tuning of the feature map normalisation that cannot be ignored. A sweet spot for parameter quantization is around 10 bits in this work, but it is also important to note that this differs based on configuration.

The primary contributing components to this work are the decrease in both the number of parameters and the number of computations needed. Adoption of BN on edge devices would be made much simpler by utilising the same arithmetic operations as a MAC on a CNN.

When comparing a BNN against a CNN, a BNN shines in overall memory footprint at a small cost to accuracy. This is further compounded by the smaller advancements in SRAM density. As such, it is important to consider the implementation of a Deep Learning Neural Network (DLNN) beyond accuracy, latency, and power consumption.

## Figures and Tables

**Table 1:** Reference Network using MNIST.

Layer Type	Output Size	Weight (1-Bit)	Parameter (32-Bit)	Memory (kB)
Convolution	26×26×8	72	8	0.04
Max-pooling	13×13×8	0	0	0
BatchNorm	13×13×8	0	32	0.12
Convolution	11×11×16	1152	16	0.2
Max-pooling	5×5×16	0	0	0
BatchNorm	5×5×16	0	64	0.24
Flatten	1×400	0	0	0
Dense	1×10	4000	10	0.53
BatchNorm	1×10	0	40	0.16
Softmax	1×10	0	0	0
Total		5224	170	1.29

**Table 2:** Network format and size using the MNIST dataset.

Network Name	Remarks	Parameter Precision	Data Format ^3^	Memory (kB)
Pico(A) ^1^	BNN, with bias and γ	32 FLP	{1,8,23}	1.29
		32 FiP ^2^	{1,8,23}	1.03
		16 FiP	{1,7,8}	0.84
		14 FiP	{1,7,6}	0.81
		12 FiP (A)	{1,7,4}	0.79
		10 FiP	{1,5,4}	0.76
Pico(B) ^1^	BNN, no bias or γ	32 FLP	{1,8,23}	1.03
		32 FiP ^2^	{1,8,23}	1.03
		16 FiP	{1,7,8}	0.84
		14 FiP	{1,7,6}	0.81
		12 FiP (A)	{1,7,4}	0.79
		12 FiP (B)	{1,5,6}	0.76
[28]	CNN, Pooling Estimation	32 FiP	-	5.35
[29]	CNN, Reduced Params, No bias	16 FiP	-	9.13
[30]	Hybrid XNOR-CNN	1b, 2b, 32 FLP	-	220.1
[31]	CNN, Hardware and Software Co-Process	16 FiP	{1,7,8}	86.8
		12 FiP	{1,5,6}	65.1

^1^ Convolution Precision at 1-Bit. ^2^ The Proposed Method (FiP) model include the operation reduction while FLP operations do not. ^3^ FiP and FLP Data Formats are represented differently, refer to Section 2.3.1 for more details.

**Table 3:** Network accuracy and statistics. Accuracy and memory is normalised against the 32-Bit Pico(A) implementation.

Network Name	Parameter Precision	Accuracy (%)	Memory (kB)	Normalised Accuracy	Normalised Memory
Pico(A)	32 FLP	96.11	1.29	1.0	1.0
	32 FiP	96.11	1.03	1.0	0.8
	16 FiP	96.15	0.84	1.0	0.65
	14 FiP	96.00	0.81	1.0	0.63
	12 FiP (A)	94.74	0.79	0.99	0.61
	10 FiP	43.18	0.76	0.45	0.59
Pico(B)	32 FLP	94.58	1.03	0.98	0.8
	32 FiP	94.58	1.03	0.98	0.8
	16 FiP	94.37	0.84	0.98	0.65
	14 FiP	92.94	0.81	0.97	0.63
	12 FiP (A)	11.35	0.79	0.12	0.61
	12 FiP (B)	55.44	0.76	0.58	0.61
[28]	32 FiP	96.3	5.35	1.0	4.1
[29]	16 FiP	97.3	9.13	1.01	7.1
[30]	1b, 2b, 32 FLP	98.4	220.1	1.02	170.6
[31]	16 FiP	98.70	86.8	1.03	67.3
	12 FiP	97.59	65.1	1.02	50.5

**Table 4:** Running on ImageNet.

Network	Method	Accuracy (Top-1%)	Parameter Utilisation ^1^ (%)	Memory Savings (kB)
MobileNet [33]	Typical	70.6	0.96	-
	Ours		-	80
XNOR-Net [34]	Typical	45.0	0.48	-
	Ours		-	0
Real-to-Binary [35]	Typical	65.0	1.29	-
	Ours		-	34

^1^ Refers to the BN (γ,β,μ,σ) and bias parameters used in the proposed simplification as a percentage of total model memory utilisation.

## Data Availability

Evaluation of the network was performed using the MNIST Dataset, which can be found at: http://yann.lecun.com/exdb/mnist/ (accessed on 2 February 2023).

## Abbreviations

The following abbreviations are used in this manuscript:

ASIC	Application Specific Integrated Circuit
BN	Batch Normalisation
BNN	Binarized Neural Network
CIFAR	Canadian Institute for Advanced Research
CNN	Convolutional Neural Network
DLNN	Deep Learning Neural Network
FC	Fully-Connected
FiP	Fixed-Point
FLP	Floating-Point
MAC	Multiply-Accumulate
MNIST	Modified National Institute of Standards and Technology
PE	Processing Element
ReLU	Rectified Linear Unit
SRAM	Static Random-Access Memory
XNOR	Exclusive NOR Gate
